# The impact of serum uric acid on biological aging and mortality risk: insights from the NHANES and CHARLS cohorts

**DOI:** 10.3389/fnut.2025.1569798

**Published:** 2025-04-22

**Authors:** Cong Zhao, Leying Zhao, Yang Liu, Li-qiao Sun, Xin-rong Li, Yaoxian Wang, Weiwei Sun

**Affiliations:** ^1^Dongzhimen Hospital, Beijing University of Chinese Medicine, Beijing, China; ^2^Department of Chinese Medicine, Cangzhou Central Hospital, Cangzhou, China; ^3^Henan University of Chinese Medicine, Zhengzhou, China

**Keywords:** serum uric acid, aging, mortality risk, CHARLS, NHANES

## Abstract

**Background:**

Serum uric acid (SUA), a byproduct of purine metabolism, exerts both antioxidant and pro-inflammatory effects, making its role in aging and chronic diseases a subject of ongoing debate. Despite this, the mechanisms by which SUA influences the aging process remain poorly understood.

**Methods:**

We analyzed data from the NHANES (1999–2010) and CHARLS (2011–2015) cohorts to investigate SUA’s impact on biological aging. Generalized linear regression models assessed SUA’s effect on biological aging markers [ΔKDM-BA, ΔPhenoAge, and allostatic load (AL)], while Cox regression models estimated its association with all-cause and premature mortality. Dose–response relationships between SUA levels and aging markers (ΔKDM-BA, ΔPhenoAge, and AL), as well as all-cause and premature mortality, were evaluated using restricted cubic splines (RCS).

**Results:**

In both cohorts, elevated SUA levels were significantly associated with accelerated aging. In the NHANES cohort, for each 1 mg/dL increase in SUA, ΔKDM-BA increased by 0.52 years (95% CI: 0.43–0.61, *p* < 0.0001), and AL increased by 0.38 (95% CI: 0.29–0.47, *p* < 0.0001). In the CHARLS cohort, SUA was similarly linked to an increase in ΔKDM-BA by 0.65 years (95% CI: 0.57–0.74, *p* < 0.0001) and AL by 0.15 (95% CI: 0.12–0.18, *p* < 0.0001). RCS analysis revealed a nonlinear association between SUA and ΔKDM-BA in NHANES, with a more pronounced acceleration of aging when SUA levels exceeded 4.16 mg/dL (nonlinear *p* < 0.0001). In CHARLS, SUA showed a nonlinear relationship with ΔKDM-BA (nonlinear *p* = 0.01). Additionally, in NHANES, SUA levels were associated with increased all-cause (HR: 1.04, 95% CI: 1.01–1.07, *p* = 0.01) and premature mortality (HR: 1.06, 95% CI: 1.00–1.13, *p* = 0.046). RCS analysis further demonstrated a U-shaped nonlinear relationship between SUA levels and both all-cause and premature mortality. In contrast, SUA did not show a significant association with mortality outcomes in the CHARLS cohort.

**Conclusion:**

Elevated SUA is associated with accelerated biological aging in both U.S. and Chinese populations, but its link to mortality was evident only in the NHANES cohort. These findings highlight SUA as a potential aging marker and call for further population-specific investigation.

## Introduction

Aging represents a progressive decline in physiological integrity (1), increasing susceptibility to chronic diseases such as cardiovascular disease (CVD) (2), metabolic disorders (3), and neurodegenerative diseases (4). Although chronological age (CA) remains a strong predictor of health risk, it inadequately reflects inter-individual variability in the aging process. In contrast, biological age (BA)—derived from multi-system physiological biomarkers—offers a more dynamic and individualized assessment of aging (5). However, the metabolic determinants underlying biological aging are not yet fully understood.

Serum uric acid (SUA), the end product of purine metabolism, has emerged as a particularly contentious factor in aging research. On one hand, SUA functions as an evolutionarily conserved antioxidant capable of scavenging reactive oxygen species (ROS) (6). On the other, elevated SUA levels can activate the NLRP3 inflammasome (7), impair endothelial function, and are linked to hypertension (8), chronic kidney disease (CKD) (9), and cardiovascular events. This biological paradox has been reflected in epidemiologic studies, many of which describe a U-shaped association between SUA levels and mortality risk (10, 11). Nevertheless, the mechanisms driving this nonlinear relationship remain unclear. Additionally, prior research has predominantly focused on single aging biomarkers or ethnically homogeneous populations, limiting both mechanistic insight and generalizability.

To address these gaps, we conducted a comparative analysis leveraging data from two nationally representative cohorts: the National Health and Nutrition Examination Survey (NHANES, 1999–2010) in the United States and the China Health and Retirement Longitudinal Study (CHARLS, 2011–2015). We applied three complementary biological aging measures—Klemera–Doubal Method Biological Age (KDM-BA), Phenotypic Age (PhenoAge), and Allostatic Load (AL)—to evaluate the associations between SUA, biological aging, and mortality outcomes. This study aims to answer three key questions: (1) Does elevated SUA accelerate biological aging independent of traditional cardiometabolic risk factors? (2) What is the association between SUA and all-cause as well as premature mortality? (3) Do specific subgroups—such as women or individuals with CKD—exhibit heightened susceptibility to SUA-associated aging effects? By integrating multidimensional aging metrics across diverse populations, this research seeks to provide robust, population-based evidence to inform more targeted strategies for SUA monitoring and intervention in aging-related health.

## Methods

### Study population and data sources

**Figure 1:**
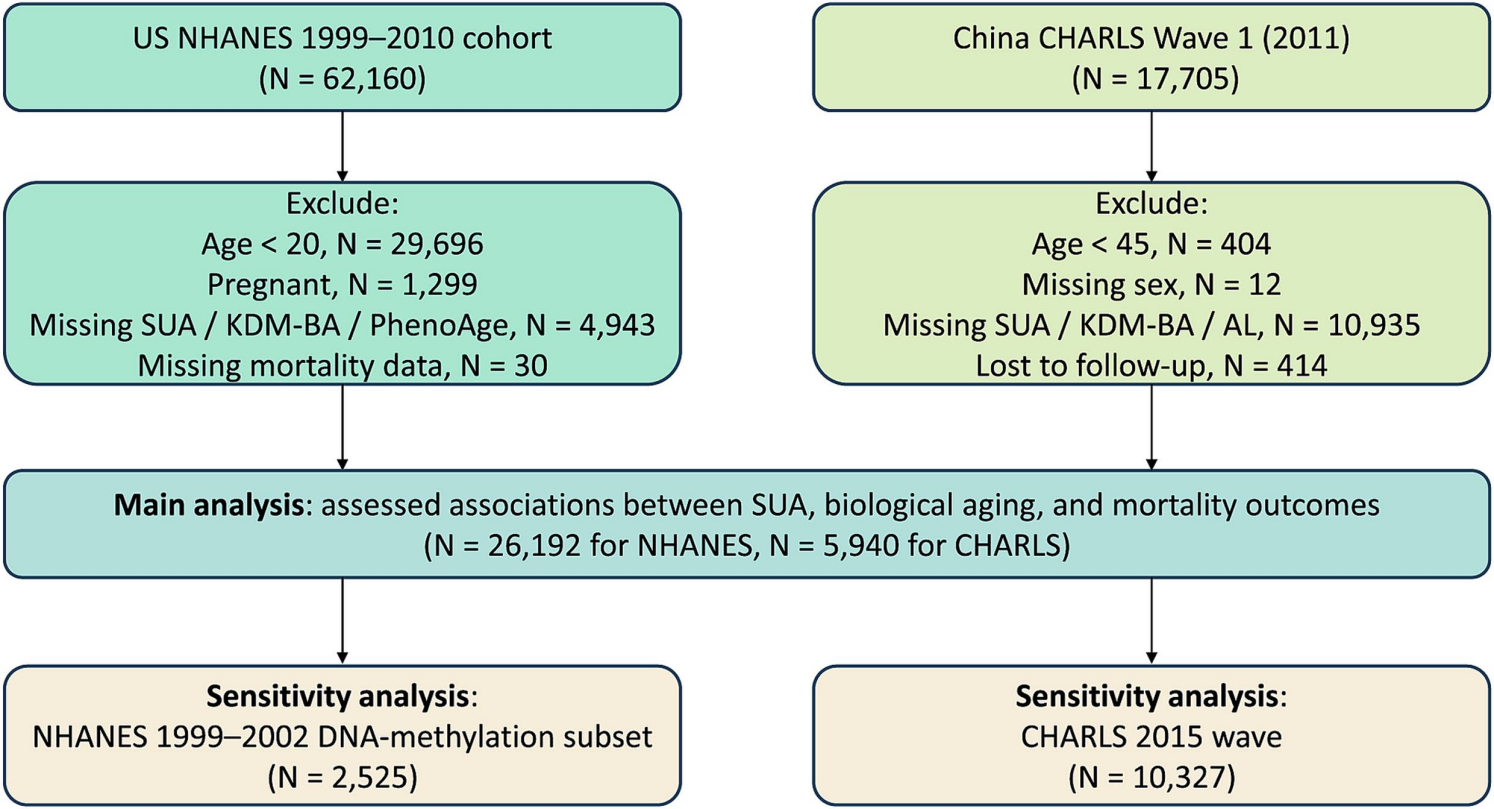
Flow diagram of participant selection.

This study is based on data from the NHANES and the CHARLS, two independent national cohorts. NHANES, organized by the National Center for Health Statistics (NCHS) under the Centers for Disease Control and Prevention (CDC), has been ongoing since the 1960s (12). It aims to collect data on health, nutrition, and lifestyle from a representative sample of the U.S. population. Detailed information on the survey and data collection methods is available on the official website.^1^ For this study, we used data from six NHANES cycles conducted between 1999 and 2010, comprising 62,160 participants. We excluded individuals under 20 years of age (*n* = 29,696), pregnant women (*n* = 1,299), those missing key biomarkers for KDM-BA or PhenoAge or SUA (*n* = 4,943), and those with incomplete mortality data as of December 31, 2019. After exclusions, 26,192 participants were included to assess the relationship between baseline SUA levels, accelerated aging, and all-cause mortality (Figure 1). All participants provided written informed consent and received ethical approval.

CHARLS is a national longitudinal survey of Chinese adults aged 45 and older (13). This study used data from the 2011 baseline (Wave 1), which included 17,705 participants. Based on study requirements, we excluded individuals under 45 years old at baseline (*n* = 404), those with missing sex information (*n* = 12), participants missing key biomarkers for KDM-BA or AL or SUA (*n* = 10,935), and those lost to follow-up during the 2013 survey (*n* = 414). After exclusions, 5,940 participants were included to assess the relationship between baseline SUA levels, accelerated aging, and all-cause mortality (Figure 1). This project was approved by the Institutional Review Board at Peking University Medical School, and all participants provided written informed consent (IRB00001052-11014 and IRB00001052-11015).

### BA measurement

To quantify aging, we utilized several previously published BA algorithms in both cohorts, including the KDM-BA, PhenoAge, and AL.

In the NHANES cohort, KDM-BA was calculated using the Klemera & Doubal method (14), which integrates eight biomarkers: hemoglobin A1c, systolic blood pressure (SBP), C-reactive protein, serum albumin, total cholesterol, alkaline phosphatase, serum creatinine, and blood urea nitrogen. PhenoAge, based on a multivariable analysis of mortality risk proposed by Levine et al. (15), uses nine clinical biomarkers—glucose, alkaline phosphatase, albumin, creatinine, C-reactive protein, white blood cell count, lymphocyte percentage, mean corpuscular volume, and red cell distribution width—to estimate an individual’s predicted age.

For the CHARLS cohort, KDM-BA was calculated using an adjusted algorithm validated for the Chinese population (16), which included total cholesterol, triglycerides, hemoglobin A1c, urea, creatinine, high-sensitivity C-reactive protein, platelet count, and SBP. Additionally, AL was used to assess cumulative physiological stress across multiple systems (17). This involved 14 biomarkers from various physiological systems, including systolic and diastolic blood pressure, body mass index (BMI), high-density lipoprotein (HDL), low-density lipoprotein (LDL), total cholesterol, triglycerides, HbA1c, fasting glucose, hemoglobin, C-reactive protein, creatinine, cystatin C, and blood urea nitrogen. Values exceeding established high-risk thresholds were assigned a score of 1, while lower values received a score of 0. The total AL score ranged from 0 to 14. KDM-BA and PhenoAge were computed using the R package BioAge^2^ (18). For each algorithm, aging acceleration was defined as ΔAge = BA − CA, where positive values indicate faster biological aging relative to chronological age. Detailed formulas and biomarker compositions for KDM-BA, PhenoAge, and AL are described in the Supplementary materials.

### Mortality outcome determination

In NHANES, mortality data through December 31, 2019, were obtained from the National Death Index, matched with death certificate records. The primary outcomes of interest were all-cause mortality and premature mortality, defined as death before age 70, based on global life expectancy in 2010 (19). In CHARLS, all-cause mortality was confirmed during the 2013 follow-up, with exact death dates recorded to calculate the interval between baseline birth dates and death dates. Premature mortality was also defined as death before age 70.

### Covariates

In the NHANES cohort, covariates included sex, race, age, marital status, BMI (<25 or ≥25) (20), education level (categorized as below high school or high school and above, based on whether participants had completed 12th grade or obtained a high school diploma), family income-to-poverty ratio (PIR: <1, 1–3, >3), smoking status (never or current smoker), and drinking habits (never, low-to-moderate, heavy), as well as disease information for hypertension, diabetes, hyperlipidemia, atherosclerotic cardiovascular disease (ASCVD), and CKD. In the CHARLS cohort, covariates included sex, age, residential area (urban or rural), geographical location (south or north), education level, marital status, drinking frequency (none, less than once a month, or once a month or more), BMI (<24 or ≥24) (21), smoking status (never or current smoker), and diagnoses for diabetes, hypertension, hyperlipidemia, CVD, and CKD. In both studies, hyperuricemia was defined as SUA levels ≥420 μmol/L (7 mg/dL) for male and ≥360 μmol/L (6 mg/dL) for female (10). Hypertension was defined as average SBP ≥ 140 mmHg, average diastolic blood pressure (DBP) ≥ 90 mmHg, or confirmation via physician diagnosis or recorded antihypertensive medication use. Diabetes was defined based on the following criteria: self-reported diagnosis, HbA1c > 6.5%, fasting glucose ≥7.0 mmol/L, random glucose ≥11.1 mmol/L, or 2-h oral glucose tolerance test (OGTT) glucose ≥11.1 mmol/L, or the use of diabetes medications or insulin (22). Hyperlipidemia was defined as triglycerides ≥150 mg/dL, total cholesterol ≥200 mg/dL, LDL-C ≥ 130 mg/dL, or HDL-C ≤ 40 mg/dL for men and ≤50 mg/dL for women; participants reporting the use of cholesterol-lowering medications were also considered to have hyperlipidemia (23). ASCVD was diagnosed if participants reported being informed by a doctor or healthcare professional that they had coronary artery disease, angina, heart attack, or stroke. The diagnostic criteria for CVD in CHARLS are based on two survey questions: “Have you been told by a doctor that you have been diagnosed with heart disease (including angina, heart attack, heart failure, coronary heart disease, or other heart problems)?” and “Have you been told by a doctor that you have been diagnosed with a stroke?.” CKD was defined by estimated glomerular filtration rate (eGFR) < 59 mL/min/1.73 m^2^ or albumin-to-creatinine ratio (ACR) > 30 mg/g (24).

### Sensitivity analysis

For the NHANES cohort, we included participants from the 1999–2002 cycles (*n* = 2,525), which uniquely provide access to DNA methylation data (Figure 1). This enabled the calculation of epigenetic biological age using two established biomarkers: Levine’s DNA methylation–based PhenoAge (DNAmPhenoAge) (15) and Horvath’s DNA methylation-predicted mortality (GrimAge) (25). These measures were derived from DNA methylation-based telomere length (DNAmTL) or quantitative polymerase chain reaction–based telomere length (qPCRTL) data and allowed for a sensitivity analysis incorporating epigenetic markers of aging. In the CHARLS cohort, we used data from the 2015 follow-up survey (*n* = 10,327) to replicate the primary analysis (Figure 1). This wave was chosen for its superior data completeness and a longer observational interval compared to the 2013 survey, thereby enhancing the robustness of aging-related inferences.

### Statistical analysis

In the NHANES cohort analysis, sampling weights, stratification, and clustering were incorporated into all analyses. Data are presented as weighted mean ± standard error (SE) for continuous variables and as unweighted frequencies with weighted percentages for categorical variables. In the CHARLS cohort, unweighted analyses were performed. Continuous variables are presented as mean ± standard deviation (SD), and categorical variables as frequencies with percentages. Generalized linear regression models were used to evaluate the relationship between baseline SUA and biological aging (ΔKDM-BA, ΔPhenoAge, AL). Cox regression models were employed to estimate the hazard ratios (HR) and 95% confidence intervals (CI) for the association between baseline SUA and all-cause mortality and premature mortality, adjusting for multiple covariates in different models. To explore the dose–response relationship between SUA and outcomes and visualize it, restricted cubic splines (RCS) were used, with percentiles set at the 10th, 50th, and 90th percentiles (26). Kaplan–Meier survival curves were used to visualize survival rates, and log-rank tests were conducted to compare survival differences between groups. Stratified and interaction analyses were performed for subgroups such as age, sex, BMI, smoking, drinking, diabetes, hypertension, hyperlipidemia, and CKD to examine their modifying effects on the main outcomes. All statistical analyses were performed using R software (version 4.4.2), with two-sided *p* < 0.05 considered statistically significant.

## Results

### Baseline characteristics

In the NHANES cohort (*n* = 26,192), the mean age was 46.85 ± 0.22 years, with females comprising 50.72%. The mean SUA level was 5.43 ± 0.01 mg/dL, with approximately 20.6% (*n* = 5,402) meeting the criteria for hyperuricemia. Individuals with hyperuricemia tended to be male, older, and former smokers, with higher rates of BMI, HbA1c, and various chronic diseases (hypertension, diabetes, hyperlipidemia, ASCVD, CKD) (Table 1). In the CHARLS cohort (*n* = 5,940), the mean age was 60.72 ± 9.84 years, with females comprising 53.41%. The mean SUA level was 4.44 ± 1.25 mg/dL, with approximately 5.4% (*n* = 323) meeting the criteria for hyperuricemia. In this cohort, individuals with hyperuricemia were also typically older, had a higher proportion of males, lived more often in urban areas, and frequently had comorbidities such as hypertension, diabetes, hyperlipidemia, ASCVD, or CKD (Table 2).

**Table 1:** Baseline characteristics of NHANES participants.

NHANES	Total (*n* = 26,192)	Without hyperuricemia (*n* = 20,790)	With hyperuricemia (*n* = 5,402)	*p* value
Age, years, mean (SE)	46.85 ± 0.22	45.86 ± 0.21	50.92 ± 0.35	< 0.0001
Sex, *n* (%)				< 0.0001
Female	12,977 (50.72)	10,615 (52.88)	2,362 (41.87)	
Male	13,215 (49.28)	10,175 (47.12)	3,040 (58.13)	
Ethnicity, *n* (%)				< 0.0001
White	13,208 (72.04)	10,352 (71.75)	2,856 (73.24)	
Black	4,885 (10.14)	3,641 (9.79)	1,244 (11.59)	
Mexican	5,335 (7.70)	4,557 (8.25)	778 (5.44)	
Other	2,764 (10.12)	2,240 (10.21)	524 (9.74)	
Marital status, *n* (%)				< 0.0001
Married/live with partner	15,776 (63.48)	12,681 (65.28)	3,095 (62.26)	
Never married	4,132 (16.31)	3,397 (16.86)	735 (15.61)	
Widowed/divorced/separated	5,874 (18.34)	4,387 (17.85)	1,487 (22.13)	
Education, *n* (%)				0.04
High school and above	18,156 (80.48)	14,430 (80.82)	3,726 (79.53)	
Below high school	7,997 (19.41)	6,328 (19.18)	1,669 (20.47)	
Alcohol user, *n* (%)				0.15
Heavy	4,932 (20.00)	3,918 (20.94)	1,014 (21.74)	
Low-to-moderate	16,339 (63.78)	12,943 (67.63)	3,396 (65.91)	
Never	3,434 (11.00)	2,712 (11.42)	722 (12.36)	
Smoke, *n* (%)				< 0.0001
Former	6,854 (25.13)	5,076 (23.71)	1778 (31.01)	
Never	13,460 (51.27)	10,879 (51.89)	2,581 (48.88)	
Now	5,853 (23.54)	4,814 (24.39)	1,039 (20.11)	
BMI, kg/m^2^, mean (SE)	28.29 ± 0.08	27.50 ± 0.07	31.59 ± 0.15	< 0.0001
Uric acid, mg/dl, mean (SE)	5.43 ± 0.01	4.94 ± 0.01	7.43 ± 0.02	< 0.0001
HbA1c, %, mean (SE)	5.51 ± 0.01	5.49 ± 0.01	5.62 ± 0.01	< 0.0001
KDM-BA, years, mean (SE)	46.32 ± 0.23	44.72 ± 0.22	52.85 ± 0.36	< 0.0001
ΔKDM-BA, years, mean (SE)	−0.54 ± 0.10	−1.14 ± 0.10	1.93 ± 0.16	< 0.0001
PhenoAge, years, mean (SE)	42.21 ± 0.25	40.66 ± 0.24	48.57 ± 0.37	< 0.0001
ΔPhenoAge, years, mean (SE)	−4.64 ± 0.08	−5.20 ± 0.08	−2.35 ± 0.12	< 0.0001
Hypertension, *n* (%)	11,071(36.00)	7,740(31.33)	3,331(55.16)	< 0.0001
Diabetes, *n* (%)	4,180 (11.18)	2,934 (9.63)	1,246 (17.56)	< 0.0001
Hyperlipidemia, *n* (%)	19,400 (72.93)	14,878 (70.21)	4,522 (84.08)	< 0.0001
ASCVD, *n* (%)	2,822 (8.03)	1872 (6.65)	950 (13.67)	< 0.0001
CKD, *n* (%)	4,911 (13.84)	3,105 (11.02)	1806 (25.96)	< 0.0001
Death during follow-up, *n* (%)	5,907 (16.25)	4,110 (14.13)	1797 (24.92)	< 0.0001
Premature death during follow-up, *n* (%)	1,313 (4.93)	947 (31.94)	366 (26.68)	0.002

Data are presented as weighted mean ± standard error (SE) for continuous variables and as unweighted frequencies with weighted percentages for categorical variables. *p*-values were calculated using survey-weighted t-tests for continuous variables and Chi-square tests for categorical variables to compare characteristics between groups. BMI, body mass index; KDM-BA, Klemera–Doubal Method Biological Age; CKD, chronic kidney disease; ASCVD, atherosclerotic cardiovascular disease.

**Table 2:** Baseline characteristics of CHARLS participants.

CHARLS	Total (*n* = 5,940)	Without hyperuricemia (*n* = 5,617)	With hyperuricemia (*n* = 323)	*p* value
Age, years, mean (SD)	60.72 ± 9.84	60.56 ± 9.80	63.34 ± 10.09	<0.0001
Sex, *n* (%)				<0.01
Female	3,148 (53.00)	3,000 (53.41)	148 (45.82)	
Male	2,792 (47.00)	2,617 (46.59)	175 (54.18)	
Marital status, *n* (%)				0.23
Married	4,919 (82.81)	4,654 (82.86)	265 (82.04)	
Never married	43 (0.72)	43(0.77)		
Widowed/divorced/separated	978 (16.46)	920 (16.38)	58 (17.96)	
Education, *n* (%)				0.92
High school and above	452 (7.62)	429 (7.64)	23 (7.12)	
Below high school	5,486 (92.39)	5,186 (92.36)	300 (92.88)	
Residence place, *n* (%)				0.02
Rural	3,892 (65.52)	3,701 (65.89)	191 (59.13)	
Urban	2048 (34.48)	1916 (34.11)	132 (40.87)	
Drink frequency last year, *n* (%)				0.07
>1/month	1,484 (24.98)	1,389 (24.73)	95 (29.41)	
<1/month	446 (7.51)	429 (7.64)	17 (5.26)	
No	4,010 (67.51)	3,799 (67.63)	211 (65.33)	
Smoke status, *n* (%)				0.21
Never	3,591 (60.46)	3,407 (60.67)	184 (56.97)	
Smoke	2,348 (39.54)	2,209 (39.33)	139 (43.03)	
BMI, kg/m^2^, mean (SD)	23.42 ± 3.92	23.37 ± 3.90	24.38 ± 4.21	<0.0001
Uric acid, mg/dl, mean (SD)	4.44 ± 1.25	4.27 ± 1.05	7.36 ± 0.96	<0.0001
HbA1c, %, mean (SD)	5.29 ± 0.81	5.29 ± 0.82	5.30 ± 0.62	0.73
KDM-BA, years, mean (SD)	60.18 ± 10.57	59.84 ± 10.48	66.08 ± 10.36	<0.0001
ΔKDM-BA, years, mean (SD)	−0.53 ± 4.27	−0.72 ± 4.16	2.73 ± 4.68	<0.0001
Allostatic load, score, mean (SD)	2.52 ± 1.75	2.44 ± 1.71	3.86 ± 1.91	<0.0001
Hypertension, *n* (%)	2,553 (42.98)	2,358 (41.98)	195 (60.37)	<0.0001
Diabetes, *n* (%)	937 (15.77)	860 (15.31)	77 (23.84)	<0.0001
Hyperlipidemia, *n* (%)	1,405 (23.65)	1,263 (22.49)	142 (43.96)	<0.0001
CVD, *n* (%)	826 (13.94)	768 (13.71)	58 (18.01)	0.04
CKD, *n* (%)	153 (2.58)	96 (1.71)	57 (17.65)	<0.0001
Death during follow-up, *n* (%)	97 (1.63)	92 (1.64)	5 (1.55)	1.00
Premature death during follow-up, *n* (%)	33 (0.56)	31 (0.55)	2 (0.62)	0.95

Data are presented as mean ± standard deviation (SD) for continuous variables and as frequencies with percentages for categorical variables. *p*-values were calculated using independent-samples t-tests for continuous variables and Chi-square tests for categorical variables to compare characteristics between groups. BMI, body mass index; KDM-BA, Klemera–Doubal Method Biological Age; CKD, chronic kidney disease; CVD, cardiovascular disease.

### Association between SUA and accelerated aging

In both cohorts, baseline SUA levels were significantly positively correlated with multiple markers of accelerated aging. In the NHANES cohort, for each 1 mg/dL increase in SUA, the difference between ΔKDM-BA increased by 1.22 years (95% CI: 1.14–1.29, *p* < 0.0001), and the difference between ΔPhenoAge increased by 0.99 years (95% CI: 0.92–1.07, *p* < 0.0001). In the CHARLS cohort, for every 1 mg/dL increase in SUA, the difference between ΔKDM-BA increased by 1.25 years (95% CI: 1.17–1.33, *p* < 0.0001) in the crude model. Similarly, in the crude model, each 1 mg/dL increase in SUA was associated with an increase of 0.34 in AL (95% CI: 0.30–0.37, *p* < 0.0001). After comprehensive adjustment for confounding factors, this association weakened but remained statistically significant (Table 3). In multivariable regression, while the effect sizes for ΔKDM-BA and AL decreased slightly after stepwise adjustments, they still indicated a stable positive association between elevated SUA levels, accelerated aging, and increased multi-system stress load.

**Table 3:** Association between SUA and accelerated aging.

NHANES	ΔKDM-BA	*P*	ΔPhenotypic age	*p*
Crude model	1.22 (1.14,1.29)	<0.0001	0.99 (0.92,1.07)	<0.0001
Model 1	1.15 (1.05, 1.24)	<0.0001	0.85 (0.76, 0.94)	<0.0001
Model 2	0.93 (0.82, 1.03)	<0.0001	0.63 (0.53, 0.72)	<0.0001
Model 3	0.52 (0.43, 0.61)	<0.0001	0.38 (0.29, 0.47)	<0.0001

CHARLS	ΔKDM-BA	*P*	Allostatic load	*P*
Crude model	1.25 (1.17,1.33)	<0.0001	0.34 (0.30,0.37)	<0.0001
Model 1	1.10 (1.01, 1.19)	<0.0001	0.42 (0.38, 0.46)	<0.0001
Model 2	1.00 (0.91, 1.09)	<0.0001	0.33 (0.30, 0.37)	<0.0001
Model 3	0.65 (0.57, 0.74)	<0.0001	0.15 (0.12,0.18)	<0.0001

For NHANES: model 1: age, ethnicity, sex, education, marital status, poverty; model 2: age, ethnicity, sex, education, marital status, poverty, BMI, alcohol user, smoke; model 3: age, ethnicity, sex, education, marital status, poverty, BMI, alcohol user, smoke, diabetes, hypertension, hyperlipidemia, atherosclerotic cardiovascular disease, chronic kidney disease. For CHARLS: model 1: sex, age, residence place, south/north, education, marital status; model 2: sex, age, residence place, south/north, education, marital status, drink frequency, smoke status, BMI; model 3: sex, age, residence place, south/north, education, marital status, drink frequency, smoke status, BMI, diabetes, hypertension, hyperlipidemia, cardiovascular disease, chronic kidney disease.

### Nonlinear relationship between SUA and accelerated aging

**Figure 2:**
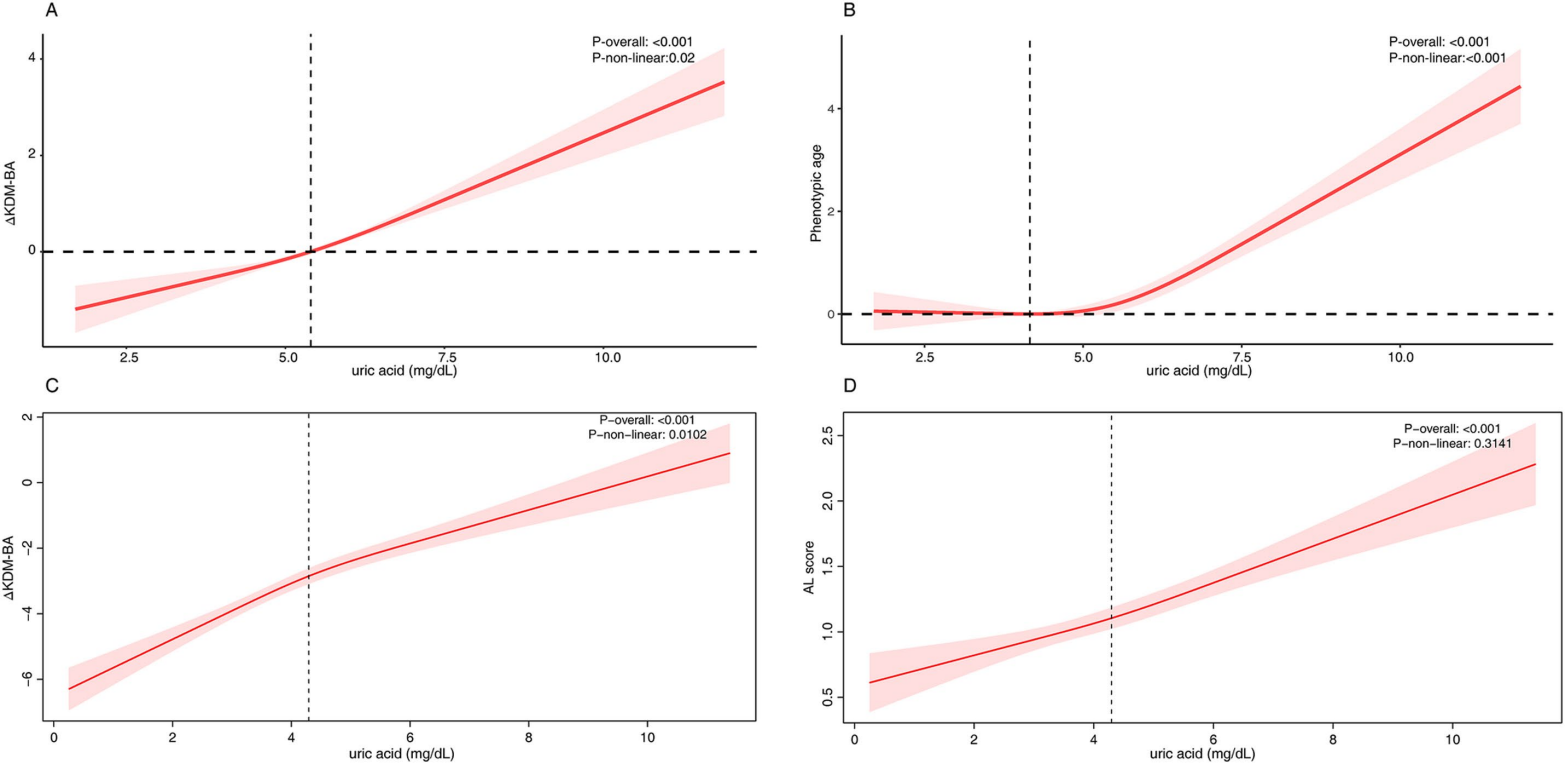
The RCS analysis between serum uric acid and accelerated aging. **(A)** NHANES - ΔKlemera-Doubal Biological Age, **(B)** NHANES - Δphenotypic age, **(C)** CHARLS - ΔKlemera-Doubal Biological Age, (C) CHARLS - allostatic load. For NHANES, the model was adjusted for age, ethnicity, sex, education, marital status, poverty, BMI, alcohol user, smoke, diabetes, hypertension, hyperlipidemia, atherosclerotic cardiovascular disease, chronic kidney disease. For CHARLS, the model was adjusted for sex, age, residence place, south/north, education, marital status, drink frequency, smoke status, BMI, diabetes, hypertension, hyperlipidemia, cardiovascular disease, chronic kidney disease.

Using RCS to assess the dose–response relationship between SUA and accelerated aging, we found a significant nonlinear association with ΔPhenoAge in the NHANES cohort (nonlinear-*p* < 0.0001), with accelerated aging effects becoming more pronounced after SUA levels exceeded approximately 4.16 mg/dL. SUA was also nonlinearly associated with ΔKDM-BA (nonlinear-*p* = 0.02) (Figures 2A,B). In the CHARLS cohort, SUA was nonlinearly associated with ΔKDM-BA (nonlinear-*p* = 0.01) and linearly associated with AL (nonlinear-*p* = 0.31) (Figures 2C,D). The overall trend showed that higher SUA levels were associated with a shift toward accelerated aging markers.

### Subgroup analysis

**Figure 3:**
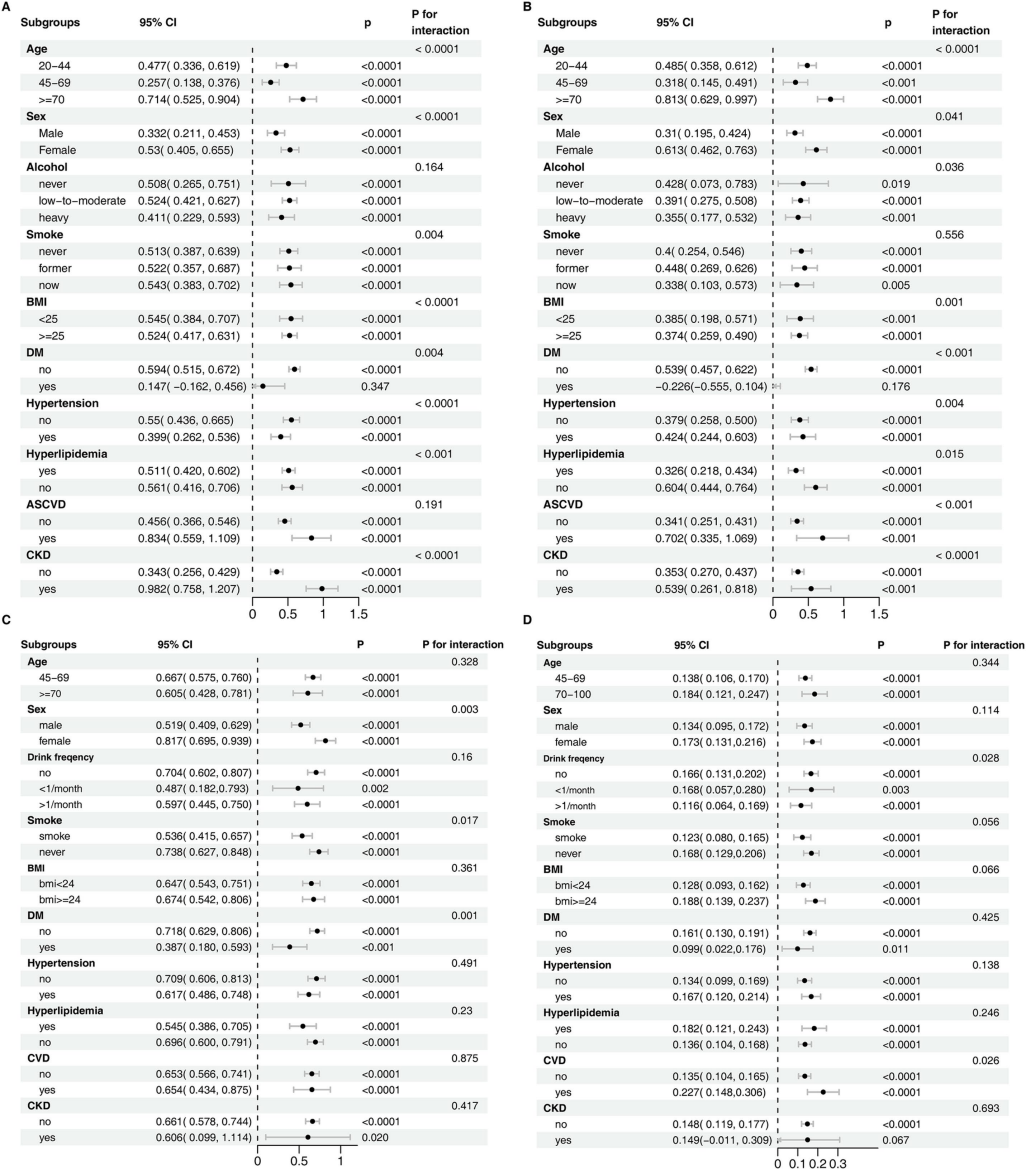
Subgroup analysis of the association between serum uric acid and accelerated aging. **(A)** NHANES - ΔKlemera-Doubal Biological Age, **(B)** NHANES - Δphenotypic age, **(C)** CHARLS - ΔKlemera-Doubal Biological Age, **(D)** CHARLS - allostatic load. For NHANES, the model was adjusted for age, ethnicity, sex, education, marital status, poverty, BMI, alcohol user, smoke, diabetes, hypertension, hyperlipidemia, atherosclerotic cardiovascular disease, chronic kidney disease. For CHARLS, the model was adjusted for sex, age, residence place, south/north, education, marital status, drink frequency, smoke status, BMI, diabetes, hypertension, hyperlipidemia, cardiovascular disease, chronic kidney disease.

After stratification by age, sex, BMI, smoking, drinking, diabetes, hypertension, hyperlipidemia, CKD, and other factors in both NHANES and CHARLS, the impact of SUA on accelerated aging markers remained consistent across most subgroups (Figures 3A–D). Some subgroups exhibited statistical interactions, suggesting that SUA had a more significant effect on accelerated aging in older adults, females and CKD patients.

### SUA and risk of all-cause and premature mortality

**Figure 4:**
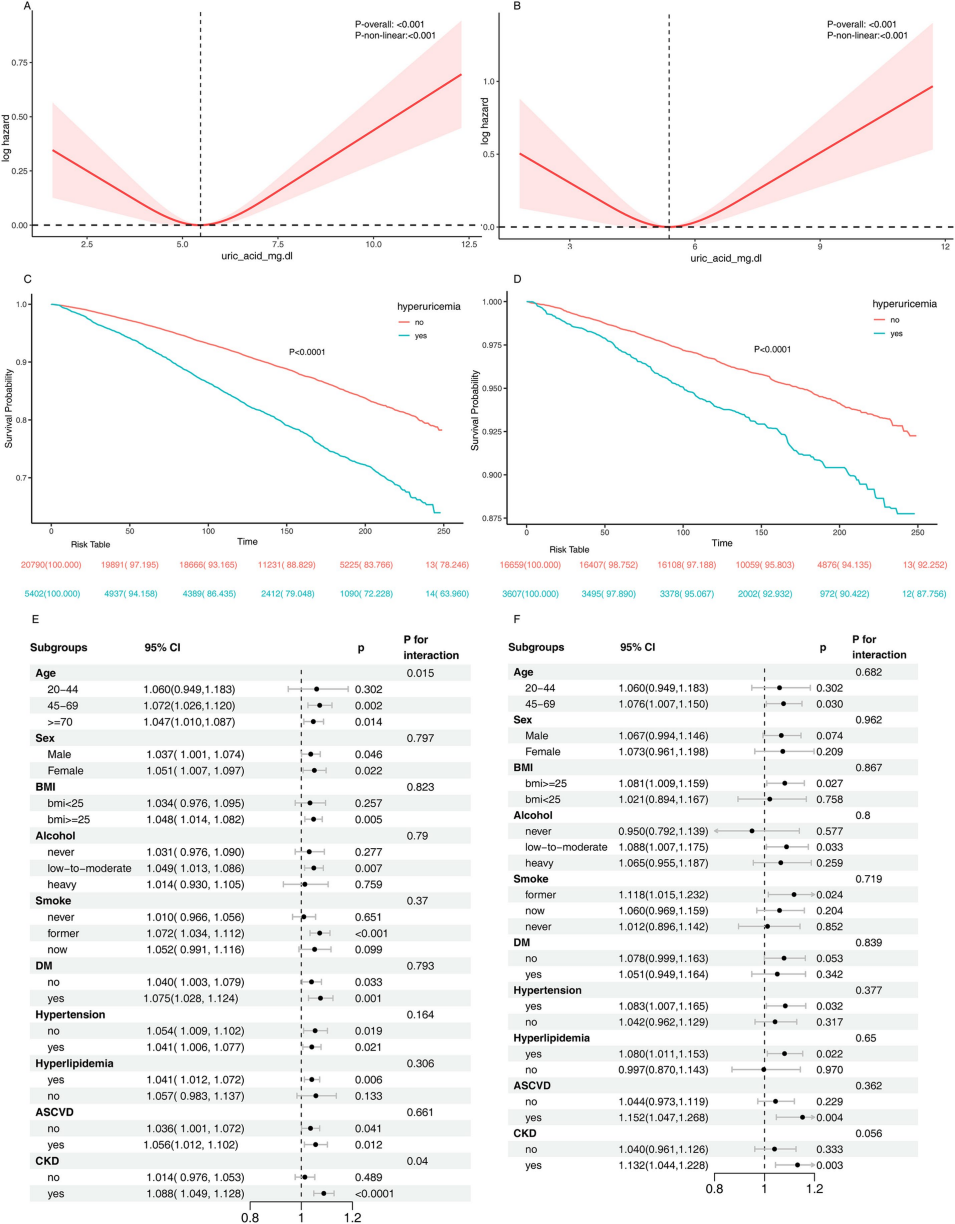
Association between serum uric acid and the risk of all-cause and premature mortality. Panel **(A,B)** show the results of RCS analysis depicting the relationship between serum uric acid levels and all-cause mortality and premature mortality, respectively. Kaplan–Meier survival curves are presented for all-cause mortality **(C)** and premature mortality **(D)**. Panel **(E,F)** display subgroup analyses evaluating the association between SUA levels and all-cause mortality and premature mortality, respectively. The model was adjusted for age, ethnicity, sex, education, marital status, poverty, BMI, alcohol user, smoke, diabetes, hypertension, hyperlipidemia, atherosclerotic cardiovascular disease, chronic kidney disease.

During long-term follow-up in NHANES (mean duration: 12.9 years), a total of 5,907 deaths were recorded, including 1,313 premature deaths. Cox regression analysis showed a statistically significant positive association between elevated SUA and both all-cause mortality and premature mortality. In the fully adjusted model, each 1 mg/dL increase in SUA was associated with a HR for all-cause mortality of 1.04 (95% CI: 1.01–1.07, *p* = 0.01), and an HR for premature mortality of 1.06 (95% CI: 1.00–1.13, *p* = 0.046) (Table 4). The restricted cubic spline results indicated a U-shaped nonlinear relationship between SUA and mortality risk (Figures 4A,B), and Kaplan–Meier survival analysis showed a higher cumulative mortality rate in individuals with hyperuricemia (Figures 4C,D). Subgroup analysis trends were consistent with the main results, with SUA having a greater impact on mortality or premature mortality in individuals with chronic diseases, although no significant interaction was observed (Figures 4E,F).

**Table 4:** Association between SUA and risk of all-cause and premature mortality.

	Death	*p*	Premature death	*p*
NHANES				
Crude model	1.23 (1.20,1.26)	<0.0001	1.202 (1.150,1.256)	<0.0001
Model 1	1.10 (1.07, 1.13)	<0.0001	1.074 (1.002,1.151)	0.044
Model 2	1.12 (1.09, 1.16)	<0.0001	1.090 (1.016,1.170)	0.016
Model 3	1.04 (1.01, 1.07)	0.01	1.064 (1.001,1.132)	0.046
CHARLS				
Crude model	1.08 (0.93,1.26)	0.30	1.07 (0.82,1.39)	0.62
Model 1	0.91 (0.77, 1.08)	0.28	0.94 (0.69, 1.28)	0.70
Model 2	0.92 (0.77,1.10)	0.36	0.93 (0.68, 1.27)	0.64
Model 3	0.90 (0.75,1.08)	0.25	0.93 (0.68, 1.27)	0.64

For NHANES: model 1: age, ethnicity, sex, education, marital status, poverty; model 2: age, ethnicity, sex, education, marital status, poverty, BMI, alcohol user, smoke; model 3: age, ethnicity, sex, education, marital status, poverty, BMI, alcohol user, smoke, diabetes, hypertension, hyperlipidemia, atherosclerotic cardiovascular disease, chronic kidney disease. For CHARLS: model 1: sex, age, residence place, south/north, education, marital status; model 2: sex, age, residence place, south/north, education, marital status, drink frequency, smoke status, BMI; model 3: sex, age, residence place, south/north, education, marital status, drink frequency, smoke status, BMI, diabetes, hypertension, hyperlipidemia, cardiovascular disease, chronic kidney disease.

In the short-term follow-up in CHARLS (mean duration: 2 years), 97 deaths and 33 premature deaths were observed. Cox regression analysis did not find a significant association between elevated SUA and all-cause or premature mortality (Table 4). In various adjusted models, the HR for all-cause mortality did not reach statistical significance. Specifically, in the crude model, each 1 mg/dL increase in SUA was associated with an HR for all-cause mortality of 1.08 (95% CI: 0.93–1.26, *p* = 0.30). After adjustment for demographic factors (age, sex, etc.), the HR decreased to 0.91 (95% CI: 0.77–1.08, *p* = 0.28). Further adjustment for lifestyle factors (BMI, smoking, drinking) yielded an HR of 0.92 (95% CI: 0.77–1.10, *p* = 0.36). In the fully adjusted model (including diabetes, hypertension, CVD, etc.), the association between SUA levels and all-cause mortality was not significant (HR = 0.90, 95% CI: 0.75–1.08, *p* = 0.25). Similarly, no significant association between SUA levels and premature mortality was observed in all models.

### Sensitivity analysis

**Figure 5:**
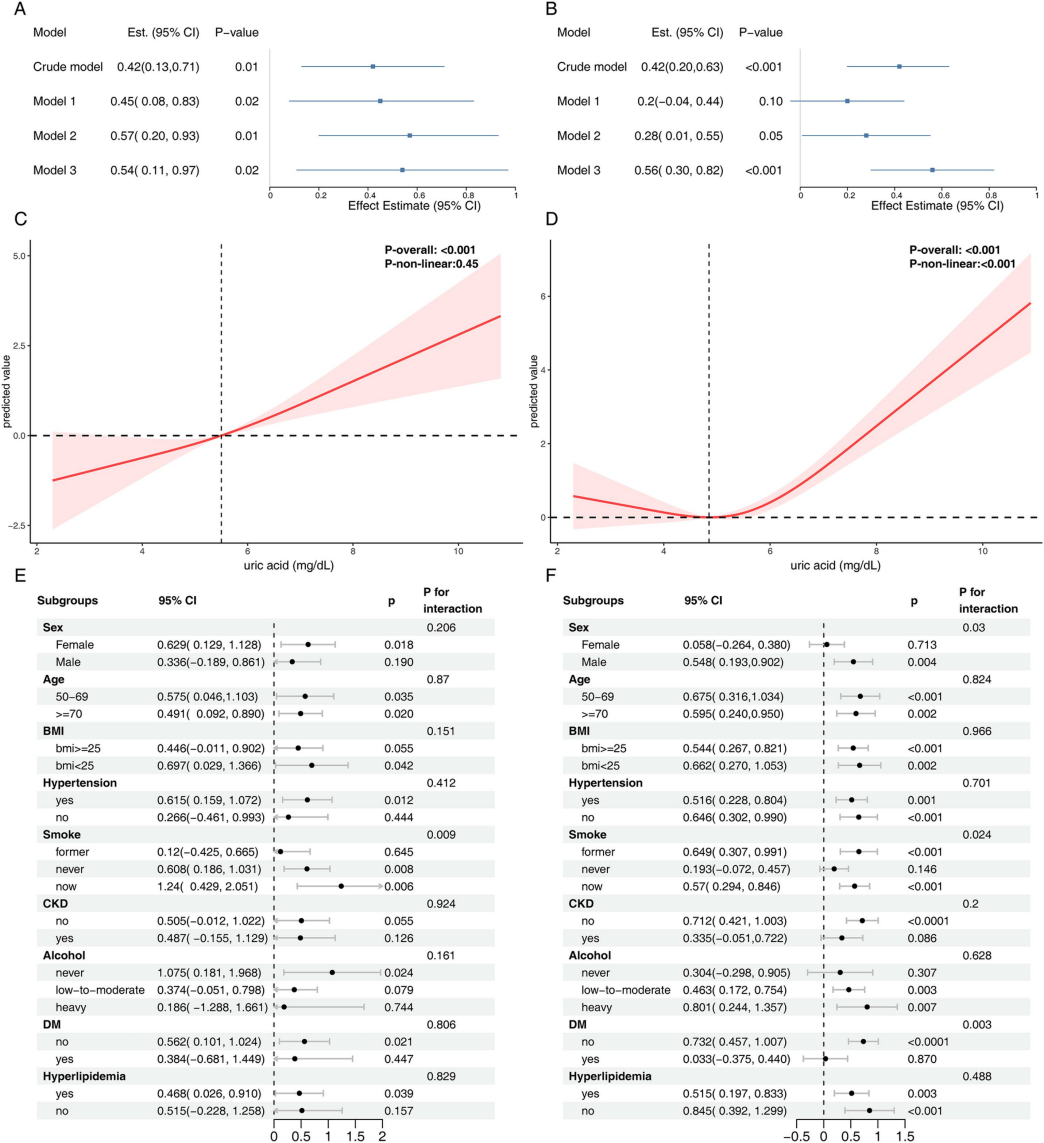
Sensitivity analysis results for the NHANES cohort. Panels **(A,B)** present the association between serum uric acid levels and ΔDNA methylation–based PhenoAge and ΔGrimAge. Panels **(C,D)** display the results of RCS analysis examining the relationship between serum uric acid and ΔDNA methylation–based PhenoAge and ΔGrimAge, respectively. Subgroup analyses of the association between serum uric acid levels and ΔDNA methylation–based PhenoAge and ΔGrimAge are shown in panel **(E,F)**. Model 1: age, ethnicity, sex, education, marital status, poverty; Model 2: age, ethnicity, sex, education, marital status, poverty, BMI, alcohol user, smoke; Model 3: age, ethnicity, sex, education, marital status, poverty, BMI, alcohol user, smoke, diabetes, hypertension, hyperlipidemia, atherosclerotic cardiovascular disease, chronic kidney disease.

**Figure 6:**
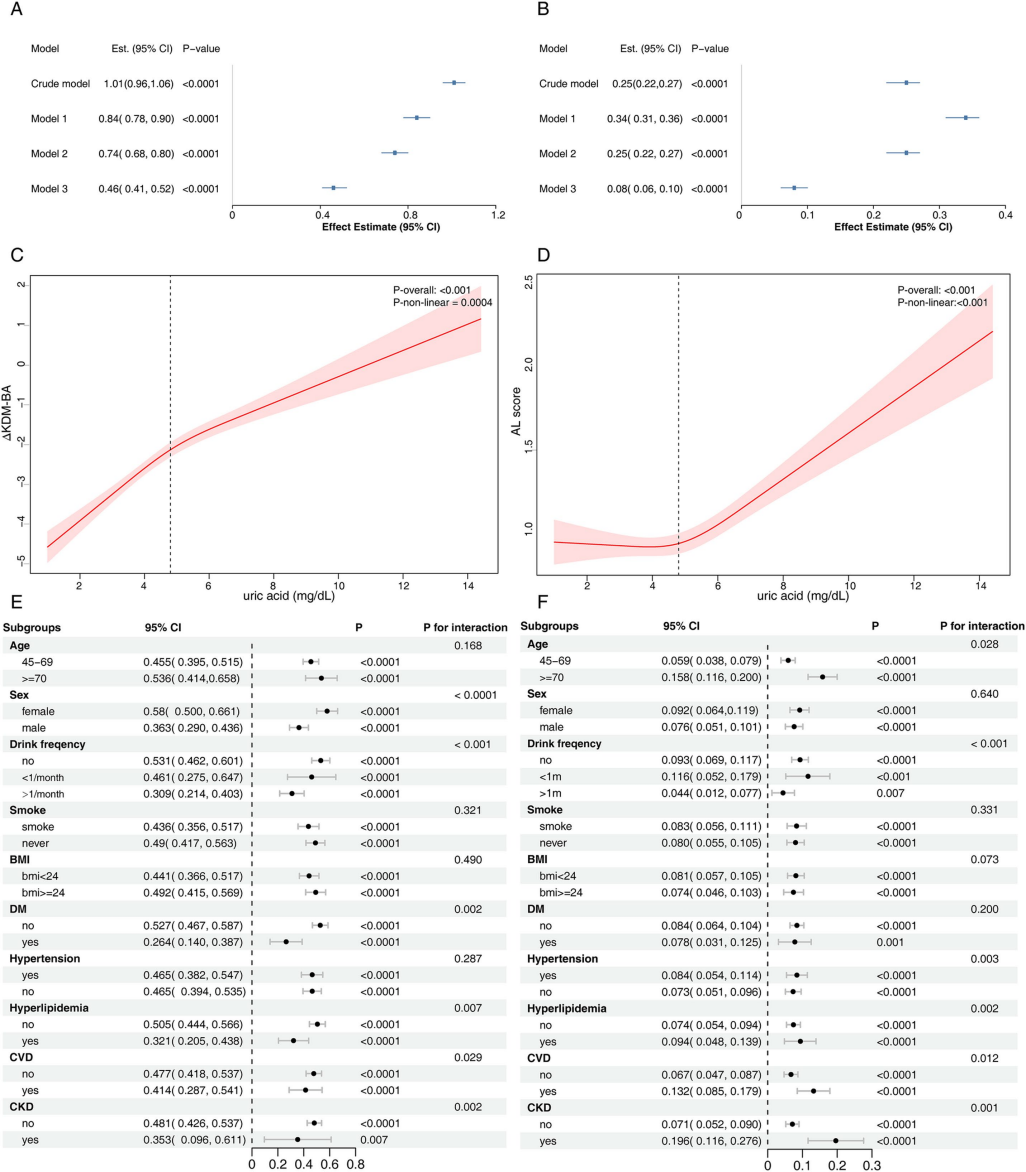
Sensitivity analysis results for the CHARLS cohort. Panels **(A,B)** present the association between serum uric acid levels and Klemera-Doubal Biological Age and allostatic load, respectively, in the CHARLS cohort. Panels **(C,D)** display the results of RCS analysis examining the relationship between serum uric acid and Klemera-Doubal Biological Age and allostatic load, respectively. Subgroup analyses of the association between serum uric acid levels and **(E)** Klemera-Doubal Biological Age and **(F)** allostatic load are shown. Model 1: sex, age, place of residence, south/north, education, marital status; Model 2: sex, age, place of residence, south/north, education, marital status, drink frequency, smoking status, BMI; Model 3: sex, age, place of residence, south/north, education, marital status, drink frequency, smoking status, BMI, diabetes, hypertension, hyperlipidemia, cardiovascular disease, chronic kidney disease.

In NHANES, a subset of participants from the 1999–2002 period was included, and DNA methylation-derived BA was recalculated (Figures 5A–F). A sensitivity analysis was conducted by including the 2015 follow-up population in CHARLS, and the results were consistent with the main analysis (Figures 6A–F). The association between SUA and aging processes remained consistent with the primary analysis, supporting the robustness of the findings.

## Discussion

Uric acid, a product of purine metabolism, has long been a subject of debate regarding its role in aging and chronic disease. Initially recognized for its antioxidant properties (27), SUA was believed to counteract oxidative stress and free radical damage, thus potentially protecting against aging and cancer (28). However, increasing evidence now suggests that elevated SUA levels may contribute to oxidative stress (29, 30), leading to cellular damage and accelerating aging. Hyperuricemia has also been strongly associated with chronic low-grade inflammation, with studies indicating that SUA can induce an inflammatory state by promoting the release of pro-inflammatory cytokines such as IL-6 and TNF-*α* (31). Additionally, hyperuricemia contributes to endothelial dysfunction (32), increases arterial stiffness (33), and heightens the risk of hypertension and CVD. Furthermore, elevated SUA levels are thought to impact telomere length and mitochondrial function, thus accelerating the aging process at the molecular level (34, 35).

Epidemiological studies have demonstrated that SUA levels are significantly correlated with several aging-related diseases, including CVD (36–38), CKD (39, 40), diabetes (41, 42), and cognitive decline (43). This study extends these findings by systematically evaluating the impact of SUA on various aging markers and mortality outcomes in two large cohorts. Consistent with prior research, we found a strong association between elevated SUA levels and accelerated biological aging (35, 43, 44). Notably, our study employed three complementary aging assessment models—KDM-BA, PhenoAge, and AL—which further confirmed that this association remained stable after adjusting for a variety of covariates. Moreover, the RCS analysis revealed a nonlinear relationship between SUA and biological aging, indicating that there is no clear “safe” threshold for SUA levels with respect to biological aging. This result was validated in two distinct national cohorts, providing robust evidence for SUA’s role in aging.

Subgroup analyses revealed that the impact of SUA on accelerated biological aging was more pronounced in women and patients with CKD. This suggests that SUA may influence the aging process through different biological mechanisms across various populations. Estrogen has been shown to lower SUA levels by inhibiting the URAT1 transporter (45), but this protective effect is lost in postmenopausal women, who are at a higher risk for gout compared to premenopausal women (46). Previous studies have indicated that hyperuricemia accelerates CKD progression through activation of the renin-angiotensin-aldosterone system (RAAS), inhibition of nitric oxide (NO) synthesis, and induction of chronic inflammation (47). Our findings align with these studies, as we observed the most significant effect of SUA on biological aging in CKD patients, with a clear interaction with all-cause mortality. This may be due to urate-induced vascular damage, oxidative stress, and immune activation. While urate-lowering therapy may help slow CKD progression, direct evidence supporting its clinical benefits for aging and mortality is lacking. Future studies should explore the underlying mechanisms and potential interventions.

The relationship between hyperuricemia, urate-lowering therapy, and mortality risk remains a topic of contention. Many observational studies have suggested a U-shaped curve between SUA levels and mortality risk (48–50), and our findings in the NHANES cohort are consistent with these studies, showing a U-shaped relationship between SUA levels and both all-cause and premature mortality. However, this relationship was not observed in the CHARLS cohort, likely due to its shorter follow-up duration (mean: 2.0 years) compared to NHANES (mean: 12.9 years), which may have reduced the statistical power to detect significant mortality associations. Notably, the average SUA in the CHARLS cohort (4.44 mg/dL) was significantly lower than in the NHANES cohort (5.43 mg/dL), and the prevalence of hyperuricemia was also much lower (5.4% vs. 20.6%). This discrepancy may be attributed to racial differences or dietary patterns, such as low-purine diets (51), though the lack of dietary data in CHARLS prevents further investigation. Previous studies have shown that gout can lead to premature death (52), but the specific impact remains unclear. This study is the first to demonstrate a significant association between hyperuricemia and premature mortality. After adjusting for confounders, the association between SUA levels and premature mortality showed a U-shaped nonlinear relationship. Janis Timsans and colleagues suggested that hyperuricemia without renal dysfunction (metabolic hyperuricemia) may increase the risk of premature death through the induction of CVD (53), a finding that is consistent with our subgroup analysis. We observed that SUA had a more pronounced effect on premature death in individuals with hypertension, hyperlipidemia, ASCVD, and CKD, emphasizing the importance of managing SUA levels in cardiovascular, renal, and metabolic diseases.

SUA levels also reflect nutritional status (54), and some studies have found that sarcopenia and weight loss are more common in patients with low SUA levels (55). Differences in body composition may help explain the association between low SUA levels and higher mortality (56). Studies conducted on hemodialysis populations found that patients with lower SUA levels, along with a low lean tissue index and high geriatric nutrition risk index, exhibited greater mortality risk. This suggests that better nutritional status, rather than elevated SUA levels, may help reduce mortality risk (57). Furthermore, the effect of ULT on mortality risk remains debated. The CARES trial showed that febuxostat treatment in gout patients with CVD resulted in significantly higher all-cause (HR = 1.22) and cardiovascular (HR = 1.34) mortality rates compared to allopurinol, with the difference being more pronounced in patients with lower baseline SUA levels (58). This suggests that excessively lowering SUA may increase mortality risk, potentially due to the loss of SUA’s antioxidant effects, acute inflammation from urate crystal dissolution, and vascular dysfunction (59). Further research is needed to clarify the causal relationship between uric acid-lowering therapy and mortality.

This study advances our understanding of SUA’s role in biological aging through several methodological improvements. By using data from two nationally representative cohorts (NHANES and CHARLS), we provide the first multi-country evidence that elevated SUA levels are consistently associated with accelerated aging across various biomarkers, including Klemera-Doubal BA, PhenoAge, and AL. These findings were robust even after adjusting for various demographic, behavioral, and confounding factors. Furthermore, restricted cubic spline analysis further described the nonlinear relationship between SUA and mortality risk in the NHANES cohort. However, this study has several limitations. First, as an observational study, it cannot establish causality, and residual confounding from unmeasured factors (e.g., dietary patterns, genetic polymorphisms in urate transporters) may still be present despite comprehensive adjustments. Second, the statistical power of the CHARLS cohort is limited due to fewer death events (*n* = 97) and a shorter follow-up period, which may have weakened our ability to detect significant mortality associations. Third, reliance on a single SUA measurement may obscure the cumulative effects of chronic hyperuricemia, as longitudinal SUA trajectories are more strongly associated with clinical outcomes than static assessments. Fourth, while the use of standardized aging biomarkers enhances comparability, differences in biomarker measurement protocols across cohorts (e.g., CRP measurement methods) may introduce heterogeneity in BA measurements. Finally, although we applied a unified definition of premature mortality across cohorts to facilitate comparison, differences in life expectancy between the U.S. and China may affect its clinical relevance (19, 60, 61). Future studies should consider cohort-specific thresholds to better reflect population-level aging and mortality risk.

## Conclusion

Based on data from the NHANES and CHARLS cohorts, we found that elevated SUA levels were significantly associated with accelerated biological aging in both populations. In the NHANES cohort, higher SUA levels were also linked to an increased risk of all-cause and premature mortality, with a U-shaped nonlinear relationship. However, this association was not observed in the CHARLS cohort, suggesting potential population-specific differences. These findings underscore the role of SUA as a potential contributor to aging and mortality risk, highlighting the need for further research to clarify the causal relationship and evaluate the long-term benefits and risks of uric acid-lowering strategies.

## Data Availability

The original contributions presented in the study are included in the article/Supplementary material, further inquiries can be directed to the corresponding author.

## Footnotes

^1^ https://www.cdc.gov/nchs/nhanes

^2^ https://github.com/dayoonkwon/BioAge
