# Supplementary material for: The impact of serum uric acid on biological aging and mortality risk: insights from the NHANES and CHARLS cohorts
Source: Front Nutr. 2025 Apr 22;12:1569798. doi: 10.3389/fnut.2025.1569798 (PMC12052535; doi:10.3389/fnut.2025.1569798)
Supplement: Supplementary file 1 [file Data_Sheet_1.PDF]

Supplementary methods 1. Calculation of KDM-BA

The Klemera–Doubal Method for estimating biological age (KDM-BA) is designed to minimize error propagation and collinearity by integrating multiple biomarkers with their respective regression parameters relative to chronological age (1). The following formula was used to compute the KDM-BA:

$$BA_{EC} = \frac{\sum_{j=1}^{m} \left(x_j - q_j\right) \frac{k_j}{s_j^2} + \frac{CA}{s_{BA}^2}}{\sum_{j=1}^{m} \left(\frac{k_j}{s_j}\right)^2 + \frac{1}{s_{BA}^2}}$$

Where:

- BA_{EC} is the estimated biological age
- x_j: observed value of biomarker j
- q_j: intercept of biomarker j regressed on chronological age
- k_j: slope of biomarker j regressed on chronological age
- s_j: standard deviation of residuals for biomarker j
- CA: chronological age
- s_{BA}: standard deviation of biological age residuals
- m: number of biomarkers used in the model

NHANES cohort:

In the NHANES cohort, KDM-BA was calculated using the original Klemera–Doubal method, incorporating eight biomarkers: hemoglobin A1c, systolic blood pressure, C-reactive protein, serum albumin, total cholesterol, alkaline phosphatase, serum creatinine, and blood urea nitrogen (BUN). Regression coefficients were derived from internal NHANES population-level models.

CHARLS cohort:

For the CHARLS cohort, an adapted KDM-BA algorithm validated for the Chinese population was used (2). This version included eight biomarkers: total cholesterol, triglycerides, hemoglobin A1c, urea, creatinine, high-sensitivity C-reactive protein (hs-CRP), platelet count, and systolic blood pressure.

Model coefficients were derived using age-stratified regressions based on CHARLS baseline participants.

Supplementary methods 2. Calculation of PhenoAge

Phenotypic Age (PhenoAge) is a composite measure of biological aging developed by Levine et al. (3), which estimates an individual's physiological age based on their risk of mortality. Unlike traditional chronological age (CA), PhenoAge is derived from a multivariate Cox proportional hazards model trained on 10-year mortality data from the U.S. NHANES III cohort, incorporating both age and selected clinical biomarkers.

PhenoAge was calculated using a weighted linear combination of nine biomarkers and chronological age, optimized to predict mortality. The biomarkers included are:

- Glucose
- Alkaline phosphatase
- Serum albumin
- Serum creatinine
- C-reactive protein
- White blood cell count
- Lymphocyte percentage
- Mean corpuscular volume
- Red cell distribution width

The composite mortality score (m) is first calculated using the following formula:

$$m = \beta_0 + \sum (\beta_i \times x_i) + \beta\_CA \times CA$$

where β_i are the coefficients derived from the original Cox regression, x_i are the individual biomarker values, and CA is chronological age.

Then, PhenoAge is calculated using the Gompertz proportional hazard transformation:

$$\text{PhenoAge} = 141.5 + \ln[\ -0.00553 \times \ln(1 - m)\ ] / 0.090165$$

Supplementary methods 3. Calculation of allostatic load

The Allostatic Load (AL) score was computed as the sum of 14 dichotomized physiological risk markers (4), each assigned a score of 1 if the value exceeded a predefined clinical cutoff and 0 otherwise. The following indicators and thresholds were used:

- Systolic blood pressure ≥ 140 mmHg
- Diastolic blood pressure ≥ 90 mmHg
- Body mass index ≤ 18.5 or ≥ 25 kg/m²
- High-density lipoprotein cholesterol < 40 mg/dL
- Low-density lipoprotein cholesterol > 160 mg/dL
- Total cholesterol ≥ 240 mg/dL
- Triglycerides ≥ 200 mg/dL
- Hemoglobin A1c ≥ 6.5%
- Fasting plasma glucose ≥ 126 mg/dL
- Hemoglobin < 13 g/dL for females or < 12 g/dL for males
- C-reactive protein > 3 mg/L
- Serum creatinine > 1.4 mg/dL
- Cystatin C > 1.03 mg/L
- Blood urea nitrogen > 20 mg/dL

References

1. Klemera P, Doubal S. A new approach to the concept and computation of biological age. *Mech Ageing Dev* (2006) 127:240–248. doi: 10.1016/j.mad.2005.10.004

2. Liu Z. Development and Validation of 2 Composite Aging Measures Using Routine Clinical Biomarkers in the Chinese Population: Analyses From 2 Prospective Cohort Studies. *J Gerontol A Biol Sci Med Sci* (2021) 76:1627–1632. doi: 10.1093/gerona/glaa238

3. Levine ME, Lu AT, Quach A, Chen BH, Assimes TL, Bandinelli S, Hou L, Baccarelli AA, Stewart JD, Li Y, et al. An epigenetic biomarker of aging for lifespan and healthspan. *Aging (Albany NY)* (2018) 10:573–591. doi: 10.18632/aging.101414

4. Ye X, Zhu D, Ding R, He P. Association of life-course socioeconomic status with allostatic load in Chinese middle-aged and older adults. *Geriatr Gerontol Int* (2022) 22:425–432. doi: 10.1111/ggi.14373
